# Refractive index of milk fat globules and extracellular vesicles in human milk

**DOI:** 10.1117/1.BIOS.3.1.012104

**Published:** 2026-02-06

**Authors:** Johanna Rebecca de Wolf, Edwin van der Pol, Chi M. Hau, Rienk Nieuwland, Nienke Bosschaart

**Affiliations:** a University of Twente, Biomedical Photonic Imaging, Faculty of Science and Technology, Enschede, The Netherlands; b Amsterdam UMC, University of Amsterdam, Biomedical Engineering & Physics, Amsterdam, The Netherlands; c Amsterdam UMC, University of Amsterdam, Laboratory of Experimental Clinical Chemistry, Laboratory Specialized Diagnostics & Research, Department of Laboratory Medicine, Amsterdam, The Netherlands

**Keywords:** breastfeeding, concentration, extracellular vesicles, human milk, milk fat globules, refractive index

## Abstract

**Significance:**

Human milk contains milk fat globules (MFGs) and extracellular vesicles (EVs). Knowing their refractive index is essential for accurate data interpretation obtained by optical methods, such as laser diffraction analysis.

**Aim:**

As the refractive index of MFGs and EVs in human milk is undescribed, we aimed to determine the refractive index of these particles and compare their refractive index values to those in bovine milk, which has been more extensively studied.

**Approach:**

Using flow cytometry, particles ranging from 200 to 650 nm were analyzed in 21 human milk samples and pooled bovine milk samples from four dairy farms.

**Results:**

Results show that the refractive index distribution of human MFGs (1.51±0.01) has a significantly higher mode compared with the distribution of bovine MFGs (1.49±0.02). In addition, EVs in human and bovine milk were best approximated by a bimodal refractive index distribution, suggesting the presence of two distinct EV populations. These populations had refractive indices of 1.38±0.01 and 1.41±0.03 for human milk, which differed from the bovine values of 1.38±0.01 and 1.42±0.03.

**Conclusions:**

This work provides insight into the refractive index of particles in human milk, which is essential for understanding milk–light interactions. Importantly, applying bovine MFG refractive index values in laser diffraction analysis for human MFGs can lead to significant errors in the estimation of particle size distributions.

Statement of Discovery This work is the first to measure the refractive index of particles in human milk, in particular, milk fat globules (MFGs) and extracellular vesicles. This knowledge is essential for accurate data interpretation in optical methods such as laser diffraction analysis, which is widely applied as a standard method for particle size analysis in milk. This study demonstrates that the refractive index distribution of human MFGs has a significantly higher mode compared with bovine MFGs.

## 1 Introduction

Human milk is a complex, heterogeneous fluid that contains different types of particles, including milk fat globules (MFGs), extracellular vesicles (EVs), casein micelles (CMs), bacteria, and cells.^1^^–^^3^ This study focuses on MFGs and EVs. MFGs are composed of a triacylglycerol core and are encapsulated by a phospholipid tri-layer.^1^^,^^4^^–^^6^ MFGs provide the infant with energy, but increasing evidence shows that MFGs also have an immunological function.^6^^–^^9^ EVs are particles encapsulated by a phospholipid membrane that contain biologically active components such as proteins and RNA.^2^^,^^10^ EVs in human milk originate from different cell types and may contribute to multiple essential functions, e.g., activation of blood coagulation,^11^ contribution to neurodevelopment,^12^ development of the infant’s immune system,^13^^–^^15^ and support of the infant’s gastrointestinal tract development.^15^ Detailed knowledge about the physical properties, composition, and concentration of the particles in human milk can help us to better understand their origin, synthesis, and biological function.

One of the physical properties of a particle includes its refractive index. Prior knowledge about the refractive index of milk particles is a prerequisite for the development and optimization of new optical technologies to analyze milk composition. Examples of these technologies include optical coherence tomography^16^^,^^17^ and photonic crystals.^18^^–^^20^ Detailed knowledge about the refractive index of milk particles is also required for adequate data interpretation of optical techniques. An example of a commonly used technique is laser diffraction analysis, which is commonly applied to analyze the particle size distribution of MFGs.^9^^,^^21^^–^^24^ In laser diffractometry, the particle size distribution is determined based on the measured diffraction pattern using Mie theory, which requires a priori knowledge of the refractive index.^25^ In the absence of data on the refractive index of human MFGs, reported studies on laser diffraction analysis of human milk rely on the refractive index of bovine MFGs.^9^^,^^21^^–^^24^ As the lipid composition of bovine and human MFGs differs significantly,^5^^,^^26^ it is likely that the refractive index of particles in human and bovine milk may differ. We have recently demonstrated that the MFG refractive index value used for laser diffraction analysis substantially affects the outcomes for the size distribution of MFGs.^23^ Therefore, the use of bovine MFG refractive index on human milk might lead to incorrect outcomes of the size distribution of MFGs.

To our knowledge, the refractive index distribution of particles such as MFGs and EVs in human milk is currently unknown. Previous studies measured the refractive index of MFGs in bovine and caprine milk by holographic imaging in combination with Lorenz–Mie theory,^27^ Abbes refractometer in combination with serial dilutions and extrapolation,^28^ refractive index-matching method,^29^ and a home-built flow cytometer in combination with simulations based on Mie theory.^30^ The advantage of flow cytometry is that a large number of single particles is analyzed, leading to an accurate and detailed refractive index distribution.^30^ In addition, commercial flow cytometers can be employed to analyze the particle size distribution and the refractive index distribution of particles with a diameter between 200 and 650 nm. This requires a sensitive forward and side scatter channel (FSC and SSC), combined with the flow cytometry scatter ratio method (Flow-SR) that was introduced by van der Pol et al.^31^ The latter method has been validated and applied for particle research in other biological fluids than milk, including extracellular vesicles in urine and blood.^32^

This study aims to quantify the refractive index distribution and concentration of scattering particles in human milk, using Flow-SR analysis. Within the particle size detection range of Flow-SR (200 to 650 nm), MFGs and EVs are the most abundant particles in terms of particle concentration compared with other milk particles such as casein micelles, bacteria and cells, or cell fragments.^2^ Knowledge on the refractive index of MFGs and EVs can help to better model and predict milk–light interactions and provides more insight into human milk particle composition. We hypothesize that the refractive index distribution of human MFGs has a higher mode compared with the refractive index distribution of MFGs in bovine milk because human milk contains more monounsaturated, polyunsaturated, and long-chain fatty acids.^33^^–^^39^ Based on the refractive index of EVs from other biological fluids than milk, we further hypothesize that the refractive index of EVs ranges between 1.36 and 1.42.^32^^,^^40^ To test these hypotheses, human milk samples from 21 donors and four pooled bovine milk samples from four dairy farms were collected and analyzed by flow cytometry. The particle refractive index distribution, particle size distribution, and concentration per sample were quantified using Flow-SR at a wavelength of 405 nm, similar to previous studies on bovine milk.^30^ In both human and bovine milk, we identified MGFs, EVs and remaining particles based on sample preparation (whole/skimmed milk), refractive index thresholds and fluorescent labelling.

## 2 Materials and Methods

### 2.1 Human Milk

Mature milk of at least 23 days postpartum was donated between June 2023 and January 2025 by 21 healthy volunteers living in The Netherlands. The exclusion criteria were lactation problems, nonsingleton pregnancies, and preterm born infants (<37 weeks). Donor information can be found in Table 1. Donors extracted milk using their own breast pump. A milk volume of 7 mL was sampled from the total extracted milk volume for analysis. The donated milk from 18 donors was kept at room temperature and prepared in the lab before freezing within 4 h after expression. The milk from three donors (donors 2, 5, and 8) was first stored in the fridge at 4°C for a maximum of 20 h before preparation. More information about milk preparation is presented in Sec. 2.3. Ethical approval was obtained from the Natural Sciences and Engineering Sciences Ethics Committee of the University of Twente (reference number 2021.118 and 230253, January 25, 2022, Enschede, The Netherlands), and all participants gave written consent prior to milk donation. The collected milk samples were also analyzed for other research purposes, as reported in Refs. 23 and 41.

**Table 1:** Milk donor specific information.

Donor number	Age donor (years)	Lactation period (months)	Time of milk expression (hh:mm)	Total extracted milk volume (mL)	Time since previous feed (h)	Sex infant	Time stored at −20°C (months)
1	34	6.5	11:00	100	11	Female	3.2
2	31	4.2	21:30	85	3.5	Male	2.8
3	37	3.2	15:15	80	1	Male	1.9
4	35	7.2	12:15	150	5	Male	2.7
5	28	5.7	16:15	105	7	Female	2.4
6	33	1.2	09:30	85	3	Male	2.4
7	31	0.8	10:10	75	5	Female	1.4
8	34	4.3	11:45	45	2.5	Female	1.4
9	30	1.3	13:30	90	3	Male	1.4
10	37	1.5	09:30	105	4	Female	0.9
11	27	5.1	12:15	130	4	Male	0.8
12	33	5.2	10:00	155	4	Male	0.5
13	39	3.7	09:30	35	2.5	Female	0.6
14	30	2.9	10:00	80	2.25	Male	0.5
15	29	4.6	14:15	27	2.25	Female	0.2
16	32	6.7	10:40	75	3.5	Male	0.6
17	31	8.5	15:45	70	7	Female	0.5
18	28	1.6	10:20	85	3.5	Female	0.5
19	30	1.9	11:00	80	4.2	Female	0.2
20	31	8.0	10:30	45	2	Female	3.4
21	29	9.9	11:45	65	3	Male	0.4
Mean ± SD	32 ± 3	4.2 ± 2.6	12:15 ± 03:00	85 ± 35	4.0 ± 2.2	—	1.4 ± 1.0

### 2.2 Bovine Milk

Four samples consisting of unprocessed bovine milk were collected in January 2025 from four different dairy farms in The Netherlands. Each farm owned approximately 100 Holstein Friesian dairy cows. Milk from all cows per farm was pooled prior to sample collection.

### 2.3 Milk Preparation and Storage

Both human and bovine milk samples were split into two aliquots. The first aliquot was stored as unprocessed, whole milk. The second aliquot was skimmed, according to the protocol described by de Wolf et al.^41^ In summary, skimming of 1240  μL milk was performed at room temperature in three centrifugation steps, namely, 200×g, 1000×g, and 3000×g for, respectively, 5, 10, and 20 min with intermediate volumes of 950 and 700  μL. The pellet and cream layer were removed in between each centrifugation step, resulting in 500  μL skimmed milk, depleted from cells and most MFGs. Whole milk and skimmed milk samples were frozen and stored at −20°C for 6 days to 3.5 months for human milk, as stated in Table 1, and a total of 7 or 14 days for bovine milk. Prior to analysis, samples were thawed at room temperature for 1 h and heated to 37°C for 1 h to dissociate MFG clusters and let CMs self-associate.^42^ Measurements were performed within 8 h after thawing.

### 2.4 Milk Dilution

Milk samples were diluted with simulated human milk ultrafiltrate (SHMUF), a buffer with a chemical composition similar to human milk serum, to optimally preserve milk particle integrity.^43^ SHMUF was prepared according to the protocol for SHMUF-Ca described by de Wolf et al.^43^ Pre-SHMUF solution was prepared by adding 0.756 g of ${\mathrm{KH}}_{2}{\mathrm{PO}}_{4}$, 0.574 g of $K_{3}\;\text{citrate}\cdot H_{2}O$, 1.01 g of ${\mathrm{Na}}_{3}\;\text{citrate}\cdot 2H_{2}O$, 0.861 g of $K_{2}{\mathrm{SO}}_{4}$, 0.311 g of ${\mathrm{MgCl}}_{2}\cdot 6H_{2}O$, 0.144 g of $K_{2}{\mathrm{CO}}_{3}$, 0.287 g of KCl, 0.0184 g of citric acid, and 39.0 g of α−D-lactose monohydrate to 1 L of demineralized water. This pre-SHMUF solution was filtered using polycarbonate Nuclepore filter discs with a pore size of 0.5  μm (Whatman, Maidstone, United Kingdom) in a 25 mm Delrin plastic syringe filter holder (Pall Corporation, Port Washington, New York, United States). The final SHMUF solution was prepared by adding 11.9 mL of 1 M ${\mathrm{CaCl}}_{2}$ per liter pre-SHMUF, and the pH was adjusted with a 1 M KOH solution to a pH of 6.7. SHMUF was used within 8 h after the addition of ${\mathrm{CaCl}}_{2}$. Chemicals ${\mathrm{KH}}_{2}{\mathrm{PO}}_{4}$, $K_{2}{\mathrm{SO}}_{4}$, $K_{2}{\mathrm{CO}}_{3}$, ${\mathrm{MgCl}}_{2}\cdot 6H_{2}O$, and ${\mathrm{CaCl}}_{2}\cdot 2H_{2}O$ were purchased from SupelCo (Merck, Rahway, New Jersey, United States) and $K_{3}\text{citrate}\cdot H_{2}O$, ${\mathrm{Na}}_{3}\;\text{citrate}\cdot 2H_{2}O$, KCl, citric acid, and α-lactose monohydrate were ordered from Sigma-Aldrich (St. Louis, Missouri, United States). All minerals were ordered in powder form.

### 2.5 Fluorescent Labeling

FITC-labeled lactadherin (Hematologic Technologies, Essex, Vermont, United States) was used as a fluorescent label. This marker binds to the phospholipid phosphatidylserine (PS) on the membrane of EVs, MFGs, and cell debris. To remove aggregates, lactadherin was centrifuged at 18,890g for 5 min at 20°C. The supernatant minus 10  μL of the starting volume was collected and used for staining. To stain, 20  μL of diluted milk was incubated with 2.5  μL of lactadherin (20.75  μg/mL) and kept in the dark for 2 h at room temperature. After the incubation, samples were diluted in 200  μL SHMUF to decrease background fluorescence from unbound reagents.

### 2.6 Flow Cytometer

The samples were analyzed using an A60-Micro flow cytometer (Apogee Flow Systems, Hemel Hempstead, United Kingdom). Particles were triggered by the 405 nm side scattering detector with a threshold of 14 a.u., corresponding to a side scattering cross section of $10\;{\mathrm{nm}}^{2}$. Lactadherin-FITC was illuminated with the 488 nm laser and detected between 500 and 550 nm. All samples were analyzed for 120 s with a flow rate of 3.0  μL/min. Swarm detection was avoided by diluting samples to achieve a count rate below 10,000 events per second. The resulting dilution factors are 300 to 15,000 times for human milk and 100,000 to 140,000 times for bovine milk.^44^ The peak value of the detected signals was used for further analysis. All details required to reproduce the flow cytometry experiments can be found in the supplemented MIFlowCyt-EV template.

### 2.7 Particle Identification

**Fig. 1:**
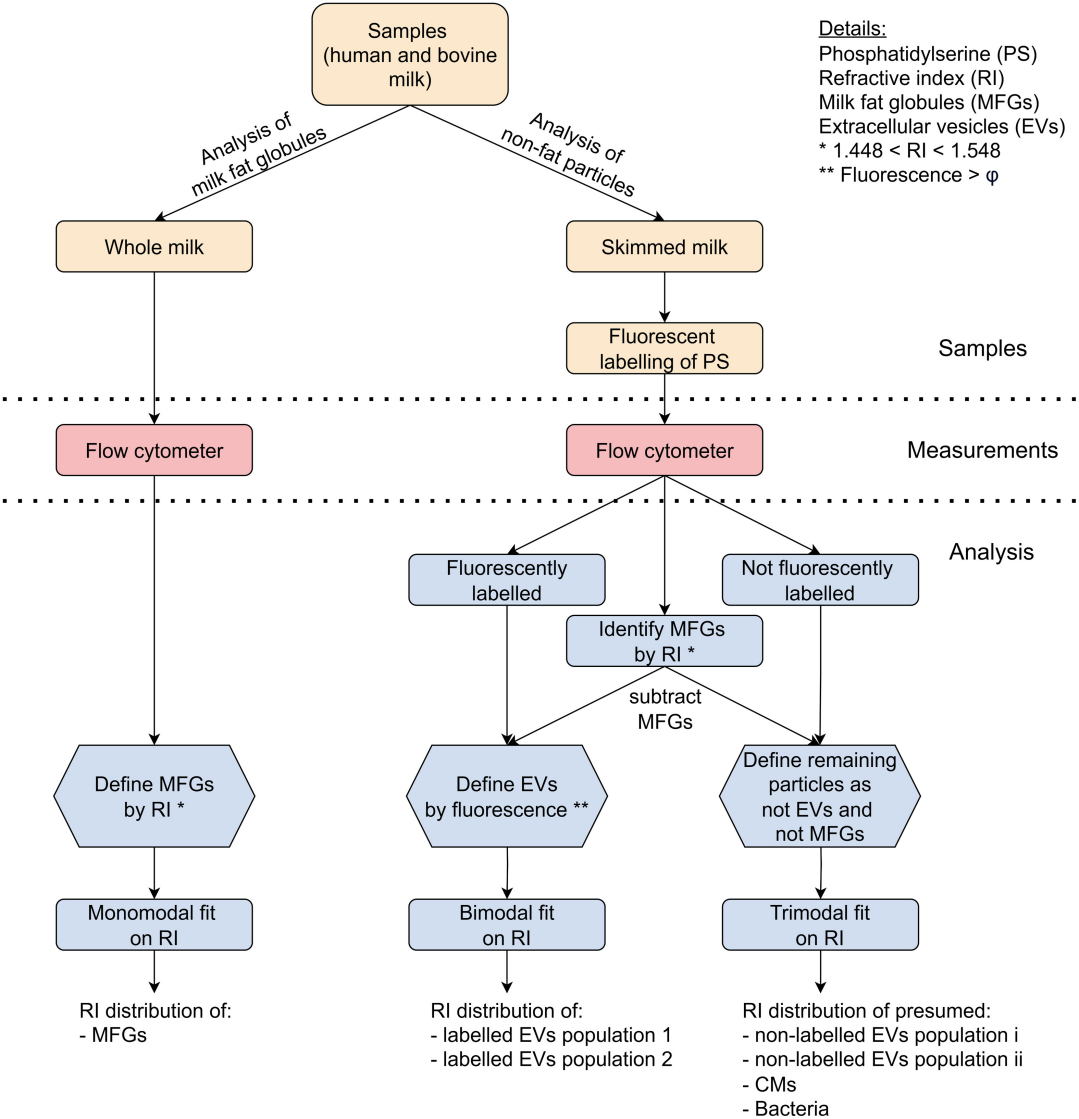
Schematic overview of the samples, measurements, and analysis steps. Acronyms are explained in the figure legend. Measurements and analysis are limited to a particle diameter range of 200 to 650 nm.

As illustrated in Fig. 1, human and bovine whole milk samples were used for MFG analysis (left), and skimmed milk samples (right) were used for measuring EVs.

In whole milk, particles with a refractive index between 1.448 and 1.548 were categorized as a distinct subgroup (Supplementary Material—MFG identification) and identified as MFGs because bovine MFGs have a refractive index of >1.44 at a wavelength of 405 nm.^30^ Only the MFGs measured within the detection limits of the refractive index analysis were analyzed, i.e., with a diameter between 200 and 650 nm. More details about the MFG selection are given in the Supplementary Material—MFG identification.

In skimmed milk, EVs were identified using fluorescent labeling of PS. We applied a total of three criteria for EV identification: (i) Particles with a fluorescent intensity above the fluorescent gate ϕ. The fluorescent gate ϕ was determined using automated fluorescent gating as described by Gankema et al.^45^ and was set between 83 and 96 MESF depending on the sample. (ii) Particles that were not identified as MFGs, and (iii) particles with a side scattering cross section between 6 and $9.6\times 10^{3}\;{\mathrm{nm}}^{2}$, as this was the detection range of the device. The remaining particles in skimmed milk that did not meet the fluorescent intensity critera and were not identified as MFGs were assumed to include unlabeled EVs, MFGs smaller than 200 nm, CMs, bacteria, and bacterial EVs.

### 2.8 Refractive Index Analysis

Flow-SR was applied to particles with a measured signal above the noise level for both the SSC and FSC.^31^^,^^32^ In summary, Flow-SR uses the ratio of side scattering over forward scattering to calculate the refractive index and diameter of particles between 200 and 650 nm. Per identified particle group, the mode and variance of the refractive index distribution were calculated using normal distribution fits, as illustrated in Fig. 1. The refractive index distributions of MFGs, EVs, and remaining particles were fitted with a monomodal, bimodal, and trimodal normal distribution, respectively. The R-squared values of the goodness of fit statistics were reported.

### 2.9 Concentration Analysis

For each particle group (i.e., MFGs, EVs, and remaining particles), the particle concentration was analyzed as a function of both sides and forward scattering cross section on a logarithmic scale with 20 bins per decade from a scattering cross section of $10^{-2}$ to $10^{6}\;{\mathrm{nm}}^{2}$. The average and standard deviation of the concentration over 21 human milk and four bovine milk samples were calculated.

### 2.10 Statistical Analysis

Statistical analyses were performed in MATLAB (R2020b). An unequal variance t-test was performed to evaluate the difference between the refractive index of particles in bovine and human milk. The Pearson correlation was calculated to evaluate how the mode refractive index per donor and per particle group correlated with the donor lactation characteristics (i.e., age of the donor, lactation period, time of milk expression, total expressed milk volume, parity, sex of the infant, and time since the previous feeding). A Pearson correlation analysis was also performed on the relation between the total particle concentration per particle group and the donor lactation characteristics. p-values below 0.05 were considered statistically significant, and p-values between 0.05 and 0.1 were considered to indicate a weak correlation.

## 3 Results

### 3.1 Refractive Index

**Fig. 2:**
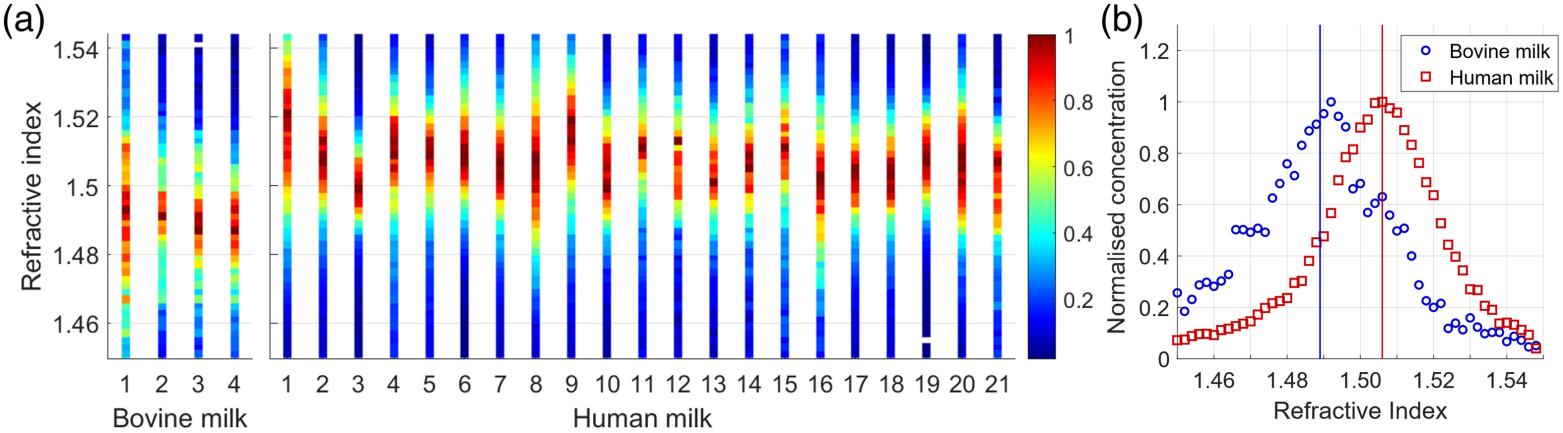
(a) Refractive index distribution at 405 nm of MFGs per whole milk sample for pooled bovine milk samples (n=4) and donated human milk samples (n=21). (b) Average refractive index distribution for MFGs in bovine (blue circles) and human (red squares) whole milk, including the mode of the monomodal normal distributions as vertical lines. Values for the refractive index modes are summarized in Table 2.

Figure 2(a) presents the refractive index distribution at 405 nm of MFGs for all bovine and human whole milk samples within the particle diameter range from 200 to 650 nm. Figure 2(b) presents the average refractive index distributions of MFGs in bovine and human whole milk. The mode and variance of the MFG refractive indices differ significantly (p<0.001) between bovine and human whole milk. The refractive index and goodness of fit of the monomodal normal distribution are given in Table 2.

**Table 2:** Refractive index of milk fat globules (MFGs), extracellular vesicles (EVs), and remaining particles in human and bovine milk with the goodness of fit values.

		Refractive index mode ± standard deviation (corresponding fraction)			
	Milk type	First population	Second population	Third population	Goodness of fit $R^{2}$
**MFGs** **(whole milk)**	**Human**	1.506 ± 0.014			0.985
	**Bovine**	1.489 ± 0.018			0.957
**EVs** **(skimmed milk)**	**Human**	1.378 ± 0.013 (41%)	1.414 ± 0.032 (59%)		0.961
	**Bovine**	1.384 ± 0.013 (32%)	1.424 ± 0.026 (68%)		0.938
**Remaining particles** **(skimmed milk)**	**Human**	1.376 ± 0.009 (37%)	1.394 ± 0.016 (33%)	1.439 ± 0.032 (20%)	0.994
	**Bovine**	1.380 ± 0.012 (0.4%)	1.399 ± 0.017 (31%)	1.424 ± 0.027 (68%)	0.997

**Fig. 3:**
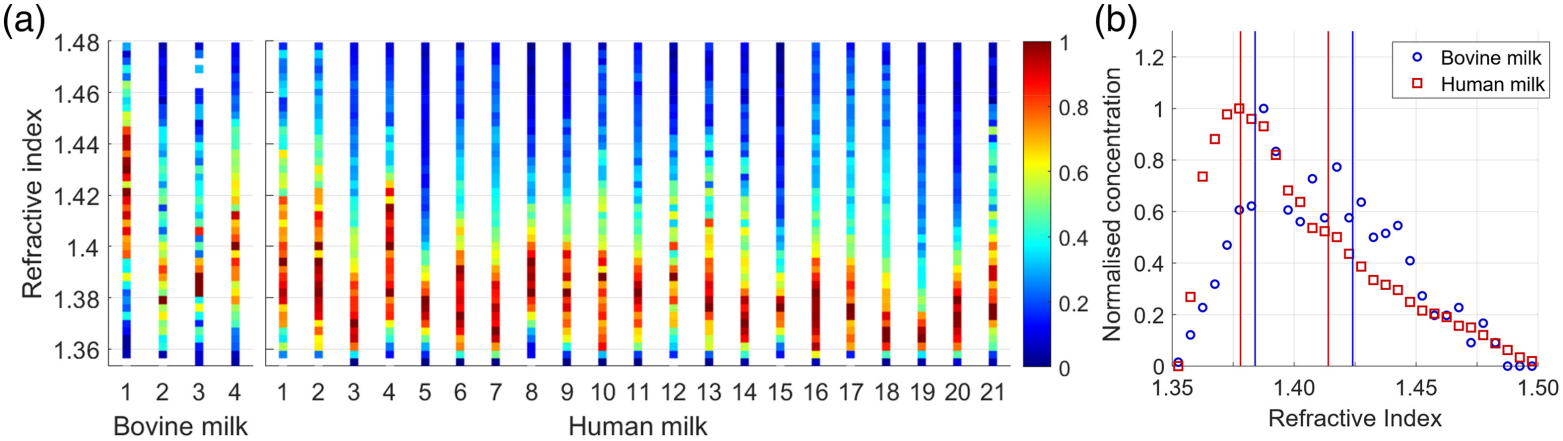
(a) Refractive index distribution of EVs per skimmed milk sample for pooled bovine milk samples (n=4) and donated human milk samples (n=21). (b) Average refractive index distribution for EVs in bovine (blue circles) and human (red squares) skimmed milk, including the modes of the bimodal normal distributions as vertical lines. Values for the refractive index modes are summarized in Table 2.

Figure 3(a) presents the refractive index distributions of EVs for all bovine and human skimmed milk samples within the particle diameter range from 200 to 650 nm. The average refractive index distributions for EVs in bovine and human skimmed milk [Fig. 3(b)] differ significantly (p<0.001). Per species, two different EV populations are retrieved through a bimodal normal distribution fit on the average refractive index distribution. The refractive index of these two EV populations in human and bovine skimmed milk is presented in Table 2.

**Fig. 4:**
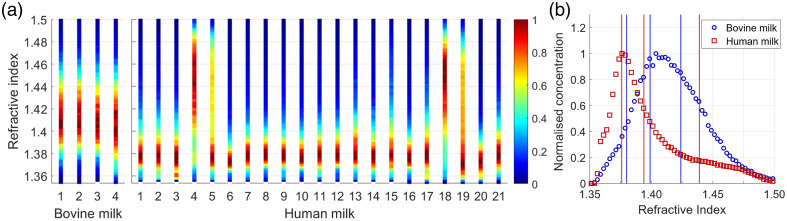
(a) Refractive index distribution of all remaining particles per skimmed milk sample for pooled bovine milk samples (n=4) and donated human milk samples (n=21). (b) The average refractive index distribution for bovine (blue circles) and human (red squares) skimmed milk, including the modes of the trimodal normal distributions as vertical lines. Values for the refractive index modes are summarized in Table 2.

Figure 4(a) presents the refractive index distribution of the remaining particles within the particle diameter range of 200 to 650 nm. This set of particles is assumed to include unlabeled EVs, CMs, and potentially bacteria and their EVs. Figure 4(b) presents the average refractive index distribution of these remaining particles for bovine and human skimmed milk, which is significantly different between bovine and human skimmed milk (p<0.001). Per species, three different particle populations are retrieved through a trimodal normal distribution fit on the average refractive index distribution. The refractive index mode and variance of these three populations per milk type are presented in Table 2. Changing the fit from a trimodal normal distribution to a bimodal or quadrimodal normal distribution would decrease the goodness of fit parameter.

### 3.2 Particle Concentration

**Fig. 5:**
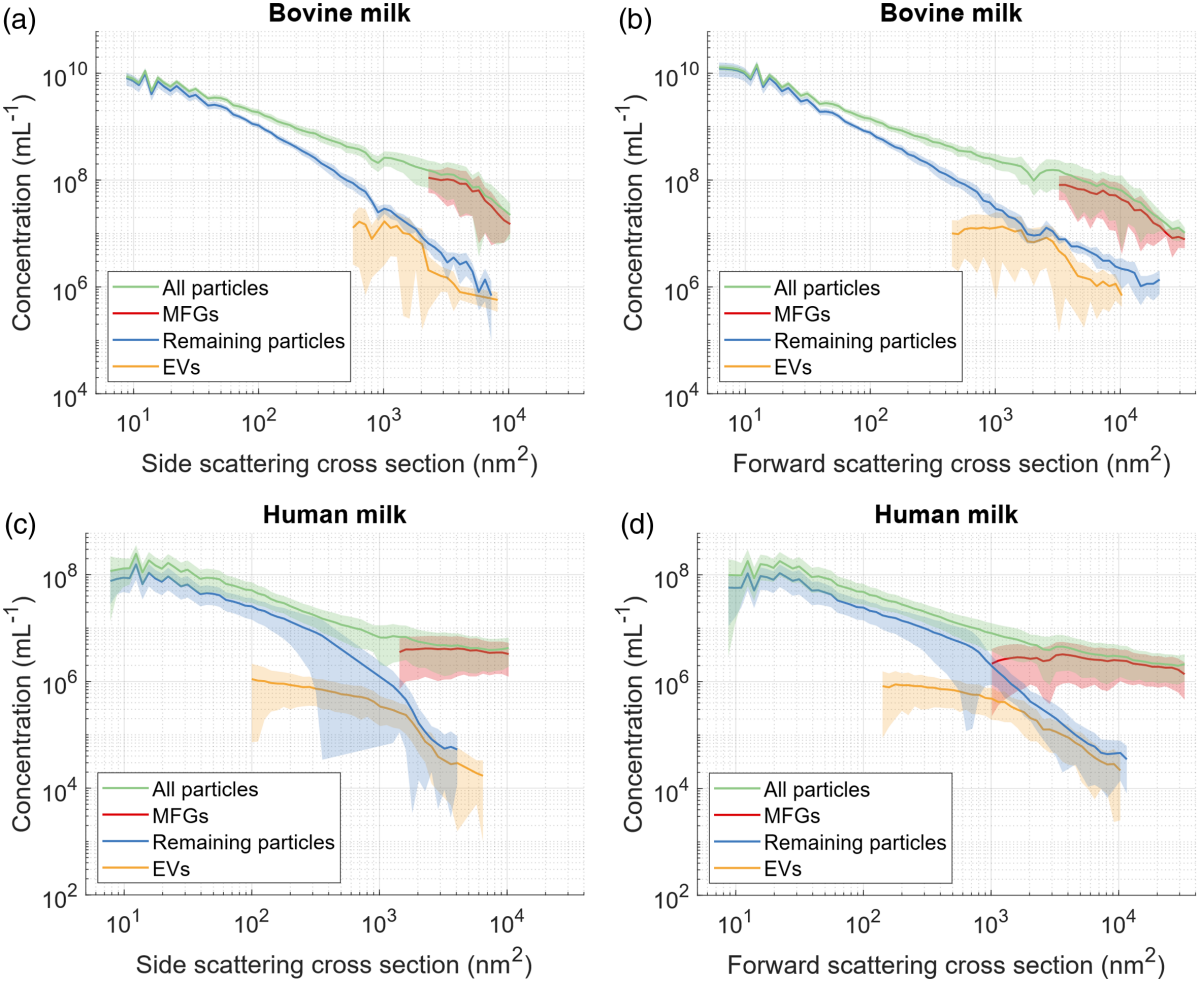
Particle concentration as a function of scattering cross section for (a) bovine milk, sideward scattering, (b) bovine milk, forward scattering, (c) human milk, sideward scattering, and (d) human milk, forward scattering. The shaded area represents the variation (standard deviation) between milk samples from different dairy farms or different donors. Used acronyms were milk fat globules (MFGs) and extracellular vesicles (EVs).

Figure 5 displays the concentration of particles as a function of the side and forward scattering cross section, in the particle diameter range from 180 to 2400 nm. The scattering cross section is a measure of how effectively incident light is scattered by a particle. It is important to note that the scattering cross section does not represent the geometrical cross section but is influenced by the combination of particle diameter and particle refractive index.^46^ For example, MFGs with a refractive index of 1.506 and a size of 250 nm have a side scattering cross section of $1050\;{\mathrm{nm}}^{2}$, whereas EVs with a refractive index of 1.378 and the same size of 250 nm have a side scattering cross section of $40\;{\mathrm{nm}}^{2}$. This also explains why Fig. 5 does not show MFGs with a side scattering cross section below $10^{3}\;{\mathrm{nm}}^{2}$.

Figure 5 reveals that the total particle concentration is about 2 orders of magnitude higher in bovine whole milk than in human whole milk. In addition, the decrease in concentration over scattering cross section is steeper for bovine whole milk than for human milk. Besides the differences between the milk samples from different species, there are also two main similarities. First, the forward scattering cross section of all measured particles is slightly higher than the side scattering cross section in this size region. Second, MFGs account for the highest concentration of particles with a side and forward scattering cross section larger than $3\times 10^{3}\;{\mathrm{nm}}^{2}$.

### 3.3 Lactation Characteristics

The relation between the mode refractive index, or concentration of identified particles in human milk and donor-specific lactation characteristics (i.e., age of the donor, lactation period, time of milk expression, total expressed milk volume, parity, sex of the infant, and time since the previous feeding) was calculated. We observed a weak correlation between three parameters: (i) The mode refractive index of MFGs and time since previous feed (p=0.059, r=0.419), (ii) the concentration of MFGs and total expressed milk volume (p=0.0697 and r=−0.404), and (iii) the concentration of remaining particles and the lactation period (p=0.0578 and r=0.420). All other p-values were above 0.1 and do not indicate any correlation.

## 4 Discussion

In this study, the refractive index distribution and concentration of human and bovine milk particles were analyzed using flow cytometry. This knowledge of particles in human milk is required for adequate interpretation of optical measurements. Furthermore, the refractive index is directly related to particle composition and can therefore reveal new insights into its biological function and origin.^47^^,^^48^

### 4.1 Refractive Index

#### 4.1.1 MFGs

As hypothesized, we observed that the refractive index distribution of MFGs in human whole milk has a higher mode than the refractive index distribution of MFGs in bovine whole milk. The mode refractive index of human MFGs was quantified as 1.506±0.014 at a wavelength of 405 nm. To the best of our knowledge, this is the first reported value for the refractive index of human MFGs. The refractive index of bovine MFGs was quantified as 1.489±0.020. This value is slightly higher than the refractive index values of bovine MFGs reported in literature at the same wavelength, e.g., 1.4743±0.0143 for raw milk,^30^ 1.4824±0.0117 for processed milk,^30^ and 1.474 for liquid commercial butter.^49^ The difference between our measured refractive index and the literature can be explained by the different methods to measure the refractive index.

The observed difference in MFG refractive index distribution between bovine and human whole milk may be explained by a difference in lipid composition between the both species. Human milk contains more long-chain fatty acids compared with bovine milk,^26^ which have a higher refractive index compared with short-chain fatty acids.^33^^,^^34^ Furthermore, the ratio of saturated and unsaturated fatty acids differs between human and bovine milk. On average, human milk consists of 40% saturated, 40% monounsaturated, and 20% polyunsaturated fatty acids relative to the total fatty acid content^35^^–^^38^^,^^47^, whereas bovine milk contains ∼75% saturated, 20% monounsaturated, and 5% polyunsaturated fatty acids.^39^

#### 4.1.2 EVs

Consistent with our hypothesis and the refractive index of EVs from other biological fluids,^32^^,^^40^ the refractive index of EVs in the diameter range of 200 to 650 nm measured in this study was between 1.36 and 1.42. We observed a significant difference in the refractive index distribution of EVs between human and bovine skimmed milk. This difference is related to compositional differences and can potentially be related to differences in biological functions of EVs between species.^2^

For both bovine and human skimmed milk, the refractive index distribution of EVs could be approximated by a bimodal distribution, suggesting the presence of two distinct EV populations. This finding can be explained by the fact that EVs can carry RNA and proteins,^10^^,^^12^^,^^50^^,^^51^ and originate from different cell types, including mammary epithelial cells, immune cells, or even bacteria.^2^^,^^15^^,^^52^ Other studies also observed heterogeneity in EVs originating from the human placenta^40^ and from human platelets.^32^ Although we did not study the cellular origin and composition of milk EVs, it does highlight the potential of flow cytometry to gain more insight into human milk EVs. In future studies, CD9, CD63, CD81, and CD86 could serve as additional markers to better characterize the origin of EVs in human milk.^15^^,^^52^^,^^53^

#### 4.1.3 Remaining particles

The group of remaining particles in this study presumably included CMs, unlabeled EVs, and a small concentration of bacteria and bacterial vesicles. A limitation of this study was that we could not differentiate between the particle types within this group. To our knowledge, literature values are lacking on the refractive index of EVs, bacteria, and bacterial EVs in either bovine or human milk, as well as the refractive index of CMs in human milk. For the refractive index of CMs in bovine milk, values were reported of 1.41 to 1.43 by Stocker et al.^49^ and 1.394 to 1.398 by Ferrer et al.^54^

In our skimmed human milk data, the refractive index distribution of the remaining particles can best be described by a trimodal normal distribution. Based on the literature values for CMs in bovine milk and the measured refractive index of labeled EVs, we hypothesize that the particles with a refractive index of 1.394±0.016 corresponded to CMs and the particles with a refractive index of 1.376±0.009 and 1.439±0.032 were groups of unlabeled EVs. By contrast, the trimodal normal distribution fit on the refractive index of bovine skimmed milk revealed that only two groups contributed. As bovine skimmed milk had a population of labeled EVs with a refractive index of 1.424±0.026, we presume that the population with a refractive index of 1.424±0.027 in bovine skimmed milk was unlabeled EVs and we presume that the group with a refractive index of 1.399±0.017 was CMs.

An interesting observation is that the refractive index distributions of the remaining particles from donors 4 and 18 are substantially different from the other donors, suggesting elevated concentrations of EVs. This observation could not be related to the documented lactation characteristics of the donors. Beyond these documented characteristics, other factors can potentially play a role in elevating human milk EV concentrations, such as maternal stress and the immunological response to viral infections.^55^

In future investigations, it is advised to differentiate among CMs, unlabeled EVs, and bacteria using fluorescent labeling and additional processing steps, such as ultracentrifugation, acidification, or addition of rennet.^2^^,^^56^ This will help to determine the refractive index of casein micelles and the different EV populations in human milk.

### 4.2 Concentration

Our data revealed a substantial concentration difference for particles in human and bovine whole milk within the sensitivity range of the flow cytometer. Bovine whole milk contained, on average, 60 times more particles than human whole milk. This difference can be partially attributed to the higher concentration of the group remaining particles, which contained among other particles CMs. The significantly higher casein concentration in bovine milk compared with human milk is in accordance with the literature.^3^

The differences in total particle concentration were also explained by the higher concentration of MFGs in bovine whole milk, compared with human whole milk. As the fat concentration in g/dL of bovine and human milk is comparable,^57^^–^^60^ the observed difference in particle concentration per milliliter can potentially be explained by differences in particle size between the MFGs of both species. As we observed a faster decrease in MFG concentration as a function of scattering cross section for bovine whole milk than for human whole milk, it can indeed be assumed that bovine whole milk contained smaller MFGs compared with human whole milk.

We further observed that MFGs had the highest concentration for a scattering cross section larger than $10^{3}\;{\mathrm{nm}}^{2}$. The combination of both a higher scattering cross section and a higher concentration makes MFGs the most dominant group of light scattering particles in both human and bovine milk.

### 4.3 Lactation Characteristics

We did not find a correlation between the mode refractive index or concentration of any particle group and the donor-specific lactation characteristics. However, we did observe three weak correlations with p-values between 0.05 and 0.1. (i) A positive relation between the mode refractive index of MFGs and the time since the previous feed. This might be related to the production of MFGs in the mammary glands.^58^ (ii) A negative relation between the particle concentration of MFGs and the expressed milk volume. A possible explanation is that the infant’s total energy intake per day is constant, but the total volume of milk produced by mothers varies.^61^ Therefore, mothers expressing larger milk volumes tend to express milk with lower fat concentrations.^62^ (iii) A positive relation between the concentration of remaining milk particles and the lactation period. This could be a result of immune particles in the milk when an infant is ill.^63^ Exposure to pathogens may be correlated with lactation period, e.g., most infants start daycare around 3 months of age in The Netherlands. Three donors with a lactation period of more than 3 months had a higher concentration of remaining particles, and one of these three donors also reported that her infant was ill at the time of milk donation.

Other donor-specific characteristics, such as genetic factors and maternal diet, may have influenced the milk particle composition or concentration. We did not register other donor-specific characteristics in this study.

### 4.4 Limitations

A limitation of using flow cytometry combined with Flow-SR to determine the refractive index distribution and particle concentration was that we could only measure the refractive index of particles between 200 and 650 nm. It is known that human and bovine milk also contain smaller and larger particles.^9^^,^^24^^,^^64^ Some MFGs in human milk have a size of 10  μm,^9^^,^^23^ and given that the majority of EVs are smaller than 200 nm,^46^ we measured only a portion of the milk EVs MFGs and EVs in milk. Future investigations could consider alternative methods to measure the refractive index of particles in a wider size ranges, such as holographic imaging,^27^ nanoparticle tracking analysis,^40^^,^^65^ or refractive index-matching method.^29^

It is also important to acknowledge that the concentration curve of all particles in whole milk contains cells and cell debris.^15^^,^^56^ Zonneveld et al.^66^ showed that cells and larger cell debris can be removed by centrifugation for 30 min at 5000×g. We also removed cells and cell debris in our skimmed milk samples, and these particles do not contribute to the group EVs or remaining particles in the skimmed milk samples.

In addition, the potential influence of active enzymes in milk on particle integrity might need to be considered. We warmed all milk samples to 37°C for 1 h after thawing to promote dissociation of MFG clusters and self-association of CMs.^42^ The digestion of MFGs in human milk by lipase is initiated in the stomach,^67^ and MFGs are therefore not affected by warming. Currently, there is limited information about enzymatic activity on EVs outside the digestive tract in the literature. CMs are stable particles in milk,^68^ and their digestion starts in the stomach.^69^ Therefore, we consider it reasonable to assume that the effect of active enzymes on the total particle concentration was minimal compared with the effect of MFG cluster dissociation and CM self-association during warming of the milk.

Another limitation of this study was that the measured concentration of EVs was an underestimation of the total concentration of EVs because not all EVs were fluorescently labeled with a fluorescent signal above the threshold, ranging between 83 and 96 MESF, depending on the sample. As mentioned above, it will be valuable for future studies to combine different fluorescent labels to include a broader range of EVs in the total EV concentration.

Last, all milk samples were frozen and thawed prior to the measurements. The influence of freezing and thawing on the refractive index has not yet been reported. We assume that this influence is negligible because the particle refractive index is dependent on particle composition, which does not alter upon freezing and thawing. The effect of freezing and thawing on the size distribution of human MFGs has been described,^21^^,^^70^^,^^71^ but the influence on the MFG particle concentration remains unknown. Based on our recent work,^43^ we can deduce that the concentration of nonfat particles in skimmed human milk decreases after freezing and thawing at 37°C for 1 h.^43^ The nonfat particle concentrations presented in the current study may therefore slightly underestimate the particle concentrations in fresh milk.

### 4.5 Impact

This study demonstrates that the refractive index distribution of human MFGs has a significantly higher mode than the refractive index distribution of bovine MFGs. This knowledge is essential for the adequate application of laser diffractometry in the analysis of human MFG size distributions. Laser diffraction analysis is widely applied as a standard method for this purpose, but the refractive index of bovine milk is commonly used for analysis in the absence of human milk values.^9^^,^^21^^,^^22^^,^^24^ Based on our measurements and the assumption that the wavelength dependency of MFGs in bovine and human milk is comparable,^29^ the refractive index for human MFGs will be 1.481 and 1.475 at laser diffractometry wavelengths of 466 and 633 nm, respectively. As we demonstrated in our previous work,^23^ this implies that the MFG size distribution from laser diffractometry shifts from a bimodal to a monomodal size distribution. This clarifies the disagreement between laser diffractometry and other methods for MFG size distribution analysis, which yield monomodal, rather than bimodal size distributions in human milk.^23^ Most importantly, these findings highlight that human milk cannot be considered a simple variation on bovine milk, and dedicated research is needed into the physical properties of human milk.

## 5 Conclusion

This study provides insight into the refractive index distribution and concentration of particles in human milk. The refractive index distribution of human MFGs has a significantly higher mode than the refractive index distribution of bovine MFGs, which is in line with the differences in fatty acid composition between the two species. This work aids in the adequate analysis of human MFG size distributions using laser diffractometry. Our results highlight that the use of bovine, instead of human MFG refractive index values in laser diffractometry, can lead to significant errors in the estimation of MFG size distributions.

This work also demonstrates that both human and bovine skimmed milk consist of two EV populations based on their refractive index distribution. In addition, human whole milk has a lower concentration of particles with a diameter between 200 and 650 nm than bovine whole milk. This knowledge of the refractive index distribution and concentration of particles in human milk contributes to a better understanding of the physical properties of human milk.

## Supplementary Material

10.1117/1.BIOS.3.1.012104.s01

## Data Availability

Data will be made available upon request.
