# Supplementary material for: Refractive index of milk fat globules and extracellular vesicles in human milk
Source: Biophotonics Discov. 2026 Feb 6;3(1):012104. doi: 10.1117/1.BIOS.3.1.012104 (PMC13052499; doi:10.1117/1.BIOS.3.1.012104)
Supplement: Supplementary file 1 [file BIOS_003_012104_SD001.pdf]

MFG identification

Figure S1a shows the refractive index and diameter of all scattering particles in whole milk from a representative milk sample of participant 2. Figure S1b shows the isolation of the MFG population from the other particles. This population is used for the further analysis of the MFGs. As can be observed in Fig S1b, the refractive index of MFGs is independent of particle size.

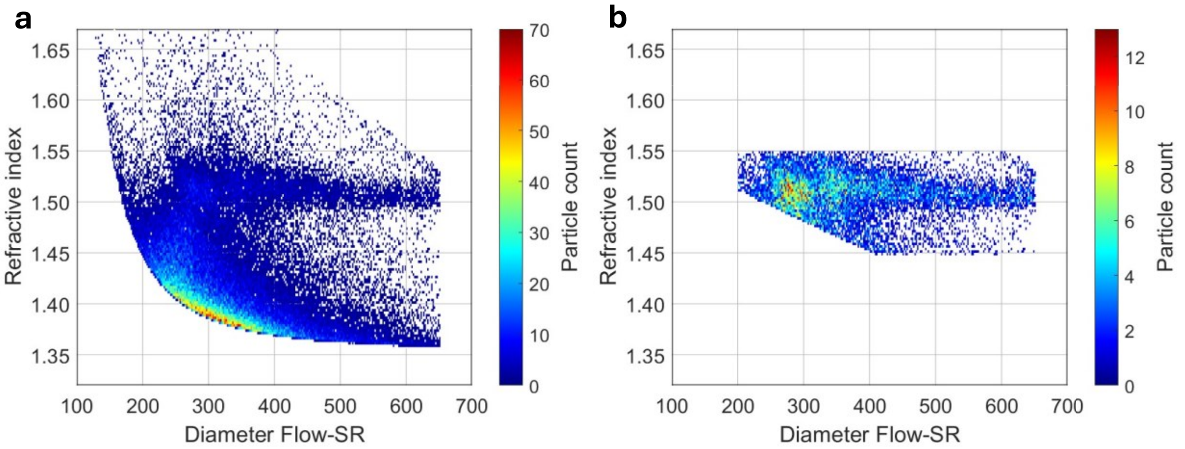

Figure S1. a Refractive index as a function of diameter for all particles in whole milk, donated by participant 2. **b** Isolated sub-population of MFG particles.

MIFlowCyt-EV of study "Refractive index of milk fat globules and extracellular vesicles in human milk"

This document aims to provide the minimum information required to reproduce the flow cytometry experiments on milk fat globules (MFGs) and extracellular vesicles (EVs) performed in the study "Refractive index of milk fat globules and extracellular vesicles in human milk". This document is based on three published standardization frameworks and guidelines [Refs. 72–74].

Contents

1 Experiment overview ... 3
1.1 Contact details ... 3
1.1.1 Experiment leader ... 3
1.1.2 Flow cytometry execution ... 3
1.2 Purpose ... 3
1.3 Keywords ... 3
1.4 Experiment variables ... 3
1.5 Experiment design and quality controls ... 4
1.6 Dates ... 4
1.7 Conclusions ... 4
2 Sample details ... 4
2.1 Sample description ... 4
2.1.1 Sample source description ... 4
2.1.2 Sample description ... 4
2.2 Sample collection ... 4
2.3 Sample storage ... 5
2.4 Sample characteristics ... 5
2.5 Sample dilution ... 5
2.6 Sample staining ... 6
2.7 Fluorescence reagents ... 8
3 Flow cytometer ... 9
3.1 Model and manufacturer ... 9
3.2 Configuration and settings ... 9
3.2.1 Flow rate and acquisition time ... 9
3.2.2 Light sources ... 9
3.2.3 Detectors ... 9
3.2.4 Trigger detector and threshold ... 9
4 Assay controls ... 10
4.1 Buffer-only controls ... 10
4.2 Buffer with reagents controls ... 10
4.3 Unstained controls ... 10
4.4 Isotype controls ... 10
4.5 Detergent treatment controls ... 11
5 Data analyses ... 11
5.1 Pulse analysis ... 11
5.2 Data sharing ... 11
5.3 Compensation details ... 11
5.4 Calibrations ... 11
5.4.1 Flow rate ... 11
5.4.2 Fluorescence calibration ... 11
5.4.3 Light scattering calibration ... 12
5.5 Gate description and boundaries ... 13

6 References... **Error! Bookmark not defined.**

1 Experiment overview

1.1 Contact details

1.1.1 Experiment leader

Name of organization	University of Twente
Address	Drienerlolaan 5<br>2522 NP<br>Enschede<br>The Netherlands
Primary contact name	Johanna R. de Wolf
Primary contact e-mail address	j.r.dewolf@utwente.nl

1.1.2 Flow cytometry execution

Name of organization	Amsterdam University Medical Centers, location AMC
Address	Meibergdreef 9<br>PO Box 22660<br>1100 DD<br>Amsterdam<br>The Netherlands
Contact name	Chi Hau
Contact e-mail address	c.m.hau@amsterdamumc.nl

1.2 Purpose

The goal of this flow cytometry experiment is to investigate the refractive index distribution and concentration of scattering particles in human milk, using flow cytometry-based Flow-SR analysis. Within the particle size detection range of Flow-SR (200-650 nm), MFGs and EVs have the most dominant share in particle concentration compared to other milk particles such as casein micelles, bacteria and cells, or cell fragments [Ref. 73]. Knowledge on the refractive index of MFGs and EVs will help to better model and predict milk-light interactions and provides more insight into human milk particle composition.

1.3 Keywords

Extracellular vesicles, flow cytometry, human breast milk, refractive index, concentration, milk fat globules, exosomes

1.4 Experiment variables

In total, 21 human milk samples and 4 pooled bovine milk samples were measured. Exclusion criteria for human participants were lactation problems, non-singleton pregnancies, and preterm born infants (< 37 weeks).

1.5 Experiment design and quality controls

All samples were measured using an autosampler, which facilitates subsequent measurements of samples in a 96-well plate. The entire study involved eight 96-well plates that was measured on eight days. The well plate contained a buffer-only control and reagents in buffer controls. Flow rate, fluorescence, and light scattering calibrations were performed daily.

1.6 Dates

Human breast milk samples were collected between June 2023 and January 2025 in the Netherlands. Bovine milk were collected in January 2025 in The Netherlands. Flow cytometry experiments were performed from 7 July 2023 till 20 October 2023 and 20 January 2025 by Chi Hau and Johanna de Wolf.

1.7 Conclusions

We concluded that the refractive index distribution of human MFGs (1.51 ± 0.01) has a significantly higher mode compared to the distribution of bovine MFGs (1.49 ± 0.02). Additionally, EVs in human and bovine milk were best approximated by a bimodal refractive index distribution, suggesting the presence of two distinct EV populations. These populations had refractive indices of 1.38 ± 0.01 and 1.41 ± 0.03 for human milk, which differed from the bovine values of 1.38 ± 0.01 and 1.42 ± 0.03. Lastly, the bovine milk contained on average 60 times more particles than human milk in the included size range.

2 Sample details

2.1 Sample description

2.1.1 Sample source description

Mature human milk was donated by healthy volunteers aged between 27 and 39, with a lactation period between 0.8 and 9.9 months postpartum, who were living in The Netherlands. Exclusion criteria were lactation problems, non-singleton pregnancies, and preterm born infants (< 37 weeks).

Unprocessed bovine milk, full-fat, were collected from four different dairy farms in the Netherlands. Each farm owned approximately 100 Holstein Friesian dairy cows.

2.1.2 Sample description

Human breast milk and bovine milk.

2.2 Sample collection

Donors extracted milk using their own breast pump between 09:30 and 21:30. The extracted milk volume ranged from 35 to 155 mL. A milk volume of 7 mL was sampled from the total extracted milk volume for analysis.

Bovine milk samples were collected from four different dairy farms. Milk from all cows per farm was pooled prior to sample collection.

Both human and bovine milk samples were split into two aliquots. The first aliquot was stored as unprocessed, whole milk. The second aliquot was skimmed, skimming of 1240 µL milk was performed at room temperature in 3 centrifugation steps, namely 200 × g, 1000 × g, and 3000 × g for

respectively 5, 10 and 20 minutes. The pellet and cream layer were removed in between each centrifugation step, resulting in 500 µL skimmed milk, absent of cells and most MFGs.

2.3 Sample storage

Human breast milk from most donors was kept at room temperature and prepared in the lab before freezing within 4 hours after expression. The milk from donors 2, 5 and 8 were first stored in the fridge for a maximum of 20 hours before preparation. Whole milk and skimmed milk samples were frozen and stored at -20 ºC between 0.2 and 3.4 months.

Bovine milk was stored at 4 ºC for a maximum of 48 hours before sample collection at the farm. Whole milk and skimmed milk samples were frozen and stored at -20 ºC between 0.2 and 0.5 months.

2.4 Sample characteristics

Unprocessed, whole milk samples are expected to contain the following particles: milk fat globules (MFGs), extracellular vesicles (EVs), casein micelles (CMs), cells, cell debris, bacteria and bacterial EVs.

Cells and cell debris were removed in our skimmed milk samples. These skimmed milk sample were expected to contain: MFGs, EVs, CMs, bacteria and bacterial EVs.

2.5 Sample dilution

As the concentration of particles in milk differs $>10^2$-fold between donors, samples require different dilutions to (1) avoid swarm detection and (2) detect a statistically significant number of events within a measurement time of a few minutes. The optimal dilution factor is the minimum dilution factor that is required to prevent swarm detection. For the flow cytometer and settings used, the optimal dilution factor for milk is $\geq 1.1 \cdot 10^2$ -fold and should result in a count rate $<1.1 \cdot 10^4$ events$\cdot$s^{-1} [Ref. 75].

To find the dilution resulting in a count rate $<1.1 \cdot 10^4$ events$\cdot$s^{-1}, we diluted each human milk sample 300 to 15,000-fold and bovine milk 100,000 to 140,000-fold in SHMUF and measured the total concentration of particles for 30 s without staining. For all experiments filtered SHMUF was used. By diluting each sample 300 to 15,000-fold and 100,000 to 140,000-fold, all samples had a count rate $<1.1 \cdot 10^4$ events$\cdot$s^{-1}. Figure S2A shows a distribution of the measured total particle concentrations of all samples in the study. Taking into account the measured concentration and flow rate, we calculated the minimum dilution factor required before staining (section 2.6) to achieve a count rate $<1.1 \cdot 10^4$ events$\cdot$s^{-1} after staining. The staining procedure adds an extra dilution factor of 11.1-fold to the overall dilution. Figure S2B shows a distribution of the applied pre-staining dilution factors of all samples in the study.

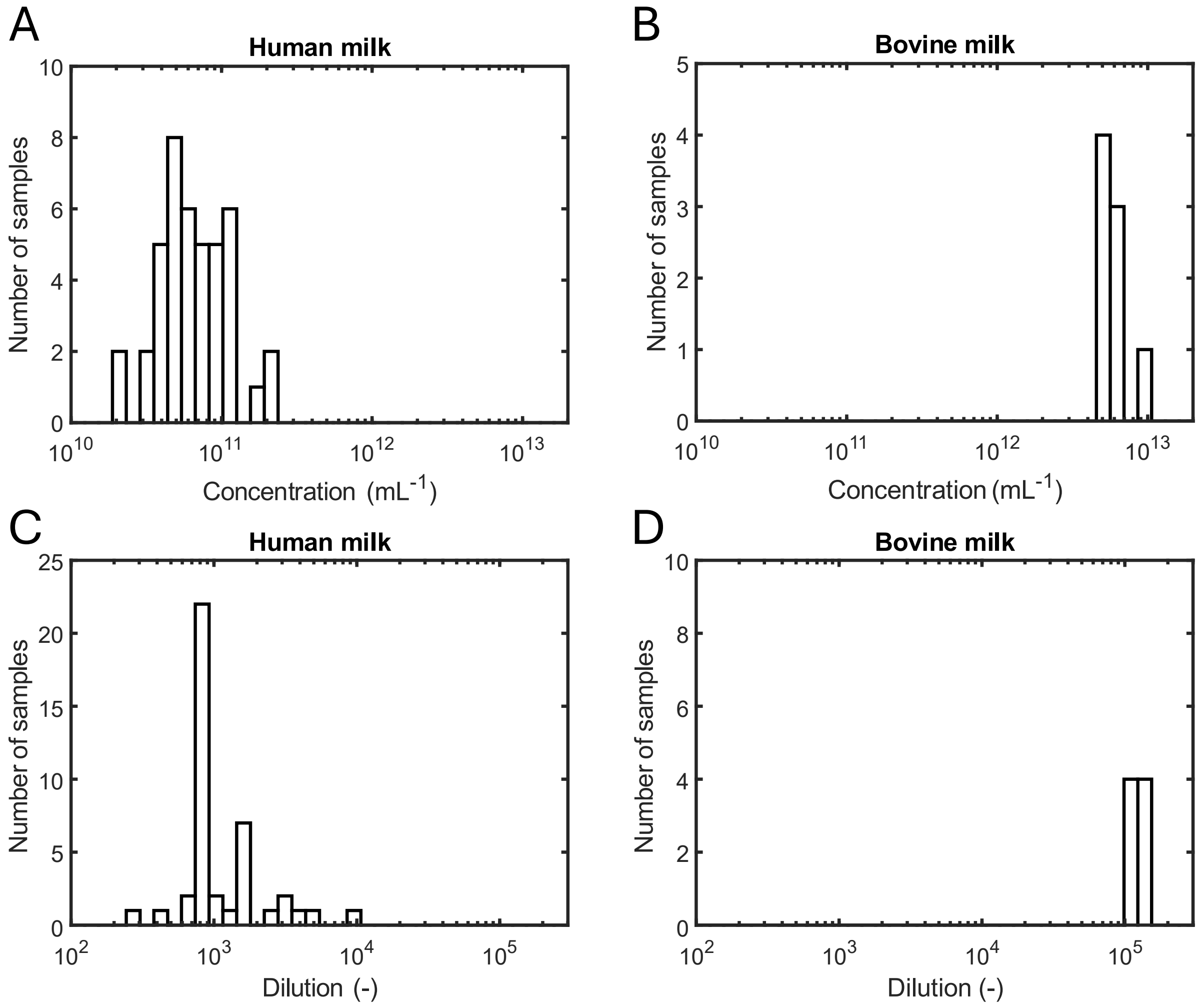

Figure S2: (A) Distribution of the total concentration of particles exceeding the trigger threshold for all human milk samples and (B) for all bovine milk samples in this study. (C) Distribution of the applied pre-staining dilution factors of all human milk samples and (D) for all bovine milk samples in the study.

2.6 Sample staining

Table S1 shows an overview of the antibodies that were used to stain EVs in milk. Prior to staining, the antibodies were diluted in SHMUF. For each antibody the optimal dilution factor was determined by titration (Table S1). To remove aggregates, diluted antibodies were centrifuged at 18,890 g for 5 min at 20 °C. The supernatant minus 10 μL of the starting volume was collected and used for staining. Each sample was stained with Lact-FITC (Fluorescein isothiocyanate). To stain, 20 μL of pre-staining diluted (

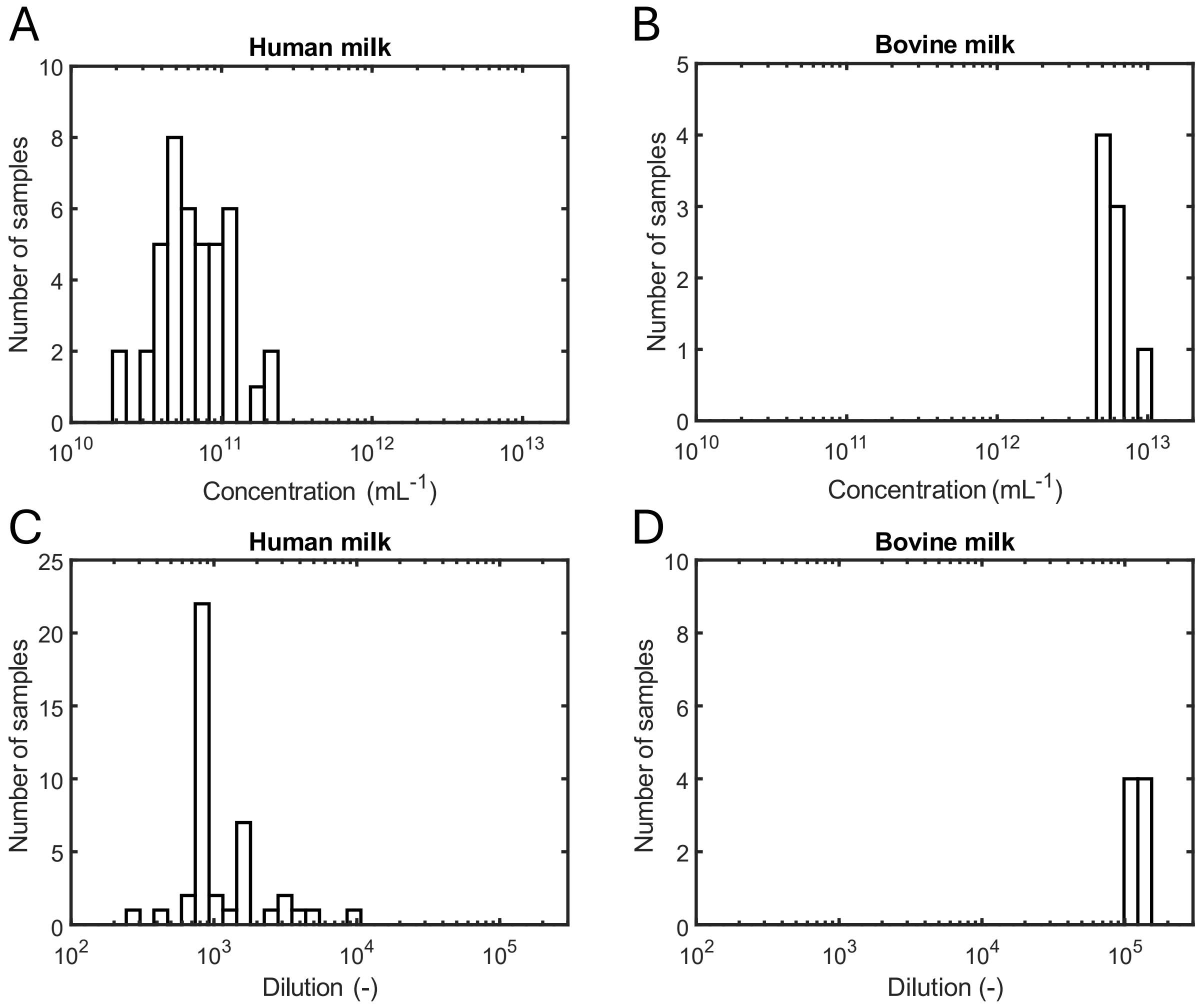

Figure S2B) milk was incubated with 2.5 µL of Lact-FITC and kept in the dark for 2 h at room temperature. After the incubation, samples were diluted in 200 µL SHMUF to decrease background fluorescence from unbound reagents.

2.7 Fluorescence reagents

**Characteristic measured**	**Analyte**	**Analyte detector**	**Reporter**	**Isotype**	**Clone**	**Concentration (μg mL^{-1})**	**Manufacturer**	**Catalog number**	**Lot number**	**Dilution factor**
Phosphatidyl serine		Lactadherin	FITC	n.a.	n.a.	83	Hematologic Technologies	BLAC-FITC	KK0804	4x

Table S1. Overview of staining reagents. Characteristics being measured, analyte, analyte detector, reporter, isotype, clone, concentration during staining, manufacturer, catalog number and lot number of used staining reagents. The concentration of staining reagents during measurements was 11.1-fold lower than the concentration during staining. FITC: fluorescein isothiocyanate.

3 Flow cytometer

3.1 Model and manufacturer

A60-Micro, Apogee Flow Systems, Hemel Hempstead, UK. The flow cytometer has not been altered. All components are original and came with the flow cytometer.

3.2 Configuration and settings

3.2.1 Flow rate and acquisition time

The flow cytometer is equipped with a syringe pump with volumetric control. Samples were analysed for 120 s at a flow rate of 3.01 μL/min.

3.2.2 Light sources

The flow cytometer has three lasers that illuminate a fixed-alignment cuvette flow cell. The adjusted powers were 100 mW, 150 mW and 150 mW for the 405-nm, 488-nm, and 638-nm laser, respectively.

3.2.3 Detectors

Table S2 shows an overview of the detectors used in this study.

Detector name	Detected property	Voltage (V)	Spectral filter bandwidth (nm)
405-SALS	Forward scattered light	470	
405-LALS	Side scattered light	367	
488-Green	FITC fluorescence	560	525/50

Table S2. Detector name, detected property, voltage and spectral filter bandwidth of the detectors used in this study. FITC: fluorescein isothiocyanate.

3.2.4 Trigger detector and threshold

The side scattering detector, operating at a wavelength of 405 nm and named 405LALS, was used as a trigger detector. The trigger threshold was set at five standard deviations above the mean background noise of the side scattering detector by fitting a normal distribution to the histogram of the background noise. The background noise was measured by running MilliQ water while triggering on the edge of the background noise of a fluorescent detector.

The channel number of the trigger threshold for milk samples that was measured from 7 July 2023 till 20 October 2023 was 24 arbitrary units (a.u.) in the data acquisition software, which corresponds to 1,536 a.u. in the datafiles due to a 6-bit mismatch between the bit-depths of the analogue-to-digital converter and the datafiles. By relating the a.u. to standard units (section 5.4.3.1), the trigger threshold corresponds to an effective side scattering cross section of 7 nm^2 and an optical diameter of 145 nm for EVs[1].

The channel number of the trigger threshold for milk samples that was measured on 20 January 2025 was 14 arbitrary units (a.u.) in the data acquisition software, which corresponds to 896 a.u. in the datafiles due to a 6-bit mismatch between the bit-depths of the analogue-to-digital converter and the datafiles. By relating the a.u. to standard units (section 5.4.3.1), the trigger threshold

[1] EVs are modelled as core-shell particles with a core refractive index of 1.38, a shell refractive index of 1.48, and a shell thickness of 6 nm.

corresponds to an effective side scattering cross section of 3 nm^2 and an optical diameter of 120 nm for EVs[2].

As this flow cytometer is equipped with semi-digital electronics, the baseline restoration and trigger threshold are applied analogically and therefore are subject to noise, which might affect the derived trigger threshold. A polystyrene bead mixture with a well-defined flat size distribution and light scattering intensities overlapping with the trigger threshold was measured to confirm that the trigger threshold corresponds to a side scattering cross section of 3 nm^2.

4 Assay controls

Assay controls recommended by the MIFlowCyt-EV framework were performed to confirm that signals originate from EVs. Fluorescence-minus-one and single-stained controls were not performed due to thorough experience with the used antibody panels and because the emission spectra of the used fluorophores do not have spectral overlap. Procedural controls were not performed because no methods to isolate EVs were applied after staining. Serial dilution control were performed on the A60-Micro for six representative plasma samples and results were published [Ref. 75]. Section 2.5 explains how swarm detection was prevented.

4.1 Buffer-only controls

Each 96-well plate contained at least 1 well with SHMUF, which was measured with the same flow cytometer and acquisition settings as all other samples. The median count rate for all SHMUF measurements was $0.17 \cdot 10^3$ events s^{-1}, which is lower than the target count rate ($7.0 \cdot 10^3$ events s^{-1}) for events in milk samples.

4.2 Buffer with reagents controls

Each 96-well plate contained a buffer with reagent control for Lact-FITC (Table S1), which was measured with the same flow cytometer and acquisition settings as all other samples. Table S3 shows a summary of the results of the buffer with reagents controls.

Reagent	Mean number of fluorescence positive events in buffer (120 s^{-1})	Mean number of fluorescence positive events in stained samples (120 s^{-1})	Mean number of fluorescence positive events in buffer / stained samples (-)
Lact-FITC	651	4641	1.40E^{-1}

Table S3. Results of the buffer with reagents controls. FITC: fluorescein isothiocyanate.

4.3 Unstained controls

Unstained controls were measured with the same dilution factor as stained milk samples. Unstained controls were used for the analysis of MFGs and the stained controls for the analysis of EVs.

4.4 Isotype controls

No isotype controls was used because Lact-FITC is not an antibody. It is a protein that bind to phosphatidylserine.

[2] EVs are modelled as core-shell particles with a core refractive index of 1.38, a shell refractive index of 1.48, and a shell thickness of 6 nm.

4.5 Detergent treatment controls

Detergent treatment controls was not performed.

5 Data analyses

To automatically apply calibrations, determine and apply gates, generate reports with scatter plots and generate data summaries, we developed and applied custom-build software (MATLAB R2020b, Mathworks, USA).

5.1 Pulse analysis

In contrast to the pulse area, the pulse height is biased towards positive fluctuations of the noise. Nevertheless, we have analyzed the pulse height for all detectors, because (1) the bias of the pulse height towards positive fluctuations of the noise is negligible within the detection range of the detectors [Ref. 76], and (2) the pulse area and height scale linearly within the detection range of the detectors, and (3) the pulse height results in higher precision than pulse area [Ref. 76].

5.2 Data sharing

Data will be made available on request.

5.3 Compensation details

No compensation was applied because no fluorophore combinations were used that have overlapping emission spectra.

5.4 Calibrations

5.4.1 Flow rate

At the start of each measurement day, we applied the automated quality control system ApoCal (#1524, Apogee Flow Systems), which checks whether the flow rate is within 20% of the adjusted flow rate of 3.01 $\mu L \cdot min^{-1}$. For all days, the flow cytometer passed this quality control check.

5.4.2 Fluorescence calibration

Calibration of the fluorescence detectors from arbitrary units (a.u.) to molecules of equivalent soluble fluorochrome (MESF) was accomplished using 2 µm FITC Quantification beads (lot 2364-85, custom-order, Becton Dickinson Biosciences). Figure S3A show the 10-base logarithm of the MESF intensities for the MESF beads versus the 10-base logarithm of the measured median fluorescence intensity of each bead population. The data are fitted with a linear function. These fluorescence calibration were used to assign MESF values for FITC population beads to rainbow beads (SPHERO™ Rainbow calibration particles, 8 peaks, 3.0-3.4 µm, lot EAP01 and lot AQ04, Spherotech). In turn, the rainbow beads, which are hard dyed beads with long-term stability, were used to apply fluorescence calibrations on a daily basis. Figure S3B show the 10-base logarithm of the assigned MESF intensities for rainbow beads versus the 10-base logarithm of the measured median fluorescence intensity of FITC population. The data are fitted with a linear function. For each measured milk sample, we added fluorescent intensities in MESF units to the flow cytometry data files using following equation:

$\mathrm{I(MESF)} = 10^{a \cdot \log_{10} \mathrm{I(a.u.)} + b}$	Equation 1

where I is the fluorescence intensity, and *a* and *b* are the slope and the intercept of the linear fits in Figure .

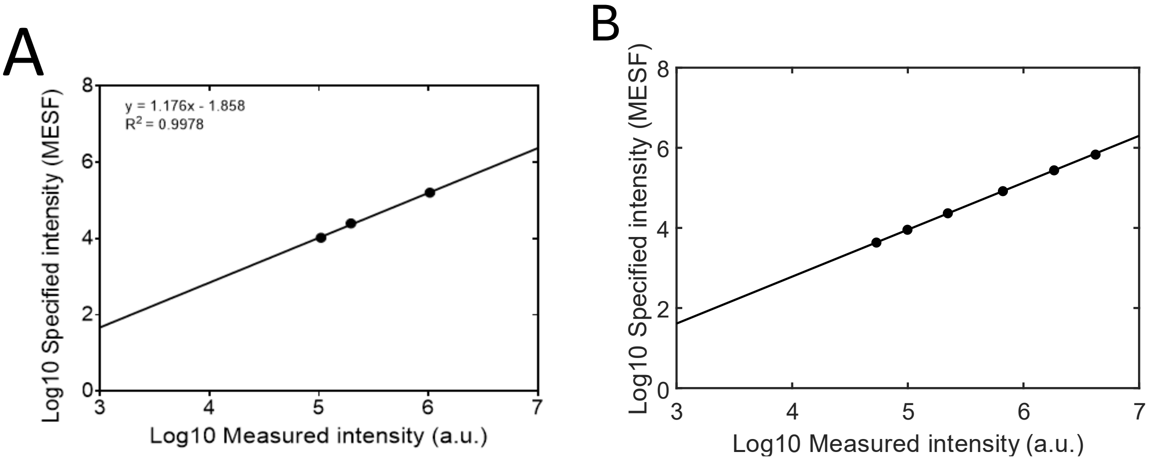

Figure S3. Calibration of the fluorescence detectors from arbitrary units (a.u.) to molecules of equivalent soluble fluorochrome (MESF). Logarithmic MESF versus logarithmic median fluorescence intensity for (A) Fluorescein isothiocyanate (FITC). Furthermore, the 10-base logarithm of the assigned MESF intensities for rainbow beads versus 10-base logarithm of the measured median fluorescence intensity of the FITC bead population are shown in (B). Data (symbols) are fitted with a linear function (line).

5.4.3 Light scattering calibration

5.4.3.1 Rosetta Calibration

Rosetta Calibration (v2.05, Exometry, The Netherlands) was used to relate the forward and side scattering intensities measured at a wavelength of 405 nm to the effective scattering cross sections [Ref. 74] and optical diameter [Ref. 75] of MFGs. MFGs are modelled as particles with a refractive index of 1.510. Figure shows print screens of the light scatter calibrations.

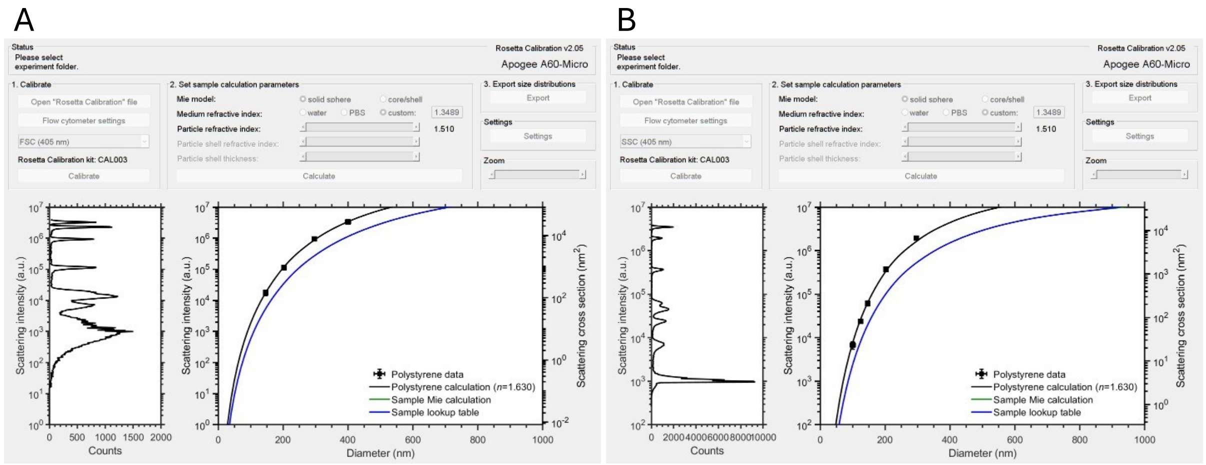

Figure S4. Forward scattering and side scattering calibration of the A60-Micro by Rosetta Calibration. To relate scatter to the diameter of MFGs, MFGs are modelled as particles with a refractive index of 1.51.

5.4.3.2 Flow Scatter Ratio (Flow-SR)

To determine the diameter and refractive index of particles and improve specificity by enabling label-free differentiation between EVs and lipoproteins, the flow scatter ratio (Flow-SR) was applied.

Flow-SR was performed as previously described [Refs. 77,78]. Lookup tables were calculated for diameters ranging from 10 to 1000 nm, with step sizes of 1 nm, and refractive indices from 1.35 to 1.80 with step sizes of 0.001. The diameter and refractive index of each particle was added to the flow cytometry datafiles by custom-build software (MATLAB R2020b).
As Flow-SR requires accurate measurements of both forward scattering and side scattering, we applied Flow-SR only to particles with diameters >200 nm, as determined by Flow-SR, and fulfilling the condition:

$\log_{10} \sigma_{SSC} > -1 \cdot \log_{10} \sigma_{FSC} + 3.6$	Equation 2

where σ_{SSC} is the side scattering cross section and σ_{FSC} is the forward scattering cross section.

5.5 Gate description and boundaries

Different gates have been applied to flow cytometry data to analyse and select milk fat globules (MFGs) and extracellular vesicles (EVs). The following gates have been applied to the flow cytometry data files by custom-build software (MATLAB R2020b).

For MFG analysis:

1. During the second that an event was measured, the count rate was within 3 standard deviations from the median count rate of the entire measurement, and…
2. the condition in Equation 2 is fulfilled, and…
   2.1.1. the refractive index, as determined by Flow-SR, is >1.448, and < 1.548, and …
   2.1.2. the diameter, as determined by Flow-SR, is between 200 nm and 650 nm.

For EV analysis:

1. During the second that an event was measured, the count rate was within 3 standard deviations from the median count rate of the entire measurement, and…
2. the condition in Equation 2 is fulfilled, and…
   2.1.1. the refractive index, as determined by Flow-SR, is < 1.448, and …
   2.1.2. the diameter, as determined by Flow-SR, is between 200 nm and 650 nm, and …
3. the side scattering cross section was between 6 nm^2 and 9.6×10^3 nm^2, and…
4. the Lact-FITC fluorescence >83 and 96 MESF depending on the sample.

The fluorescence gates, which differentiate positively stained particles from background noise, were automatically determined with a publicly available MATLAB script using a tuning factor of 2 [Ref. 76].
